# One-stage posterior-only approach in surgical treatment of single-segment thoracic spinal tuberculosis with neurological deficits in adults: a retrospective study of 34 cases

**DOI:** 10.1186/s12891-015-0640-0

**Published:** 2015-08-05

**Authors:** Hao Zeng, Penghui Zhang, Xiongjie Shen, Chengke Luo, Zhengquan Xu, Yupeng Zhang, Zheng Liu, Xiyang Wang

**Affiliations:** Department of Spine Surgery, the Xiangya Hospital of Central South University, 87# Xiangya Road, Changsha, Hunan 410008 People’s Republic of China; Department of Spine Surgery, Hunan Provincial People’s Hospital, Changsha, Hunan 410005 People’s Republic of China

**Keywords:** Spinal tuberculosis, Single-segment, Neurological deficits, Posterior-only approach, Thoracic

## Abstract

**Background:**

There are quite a few controversies on the surgical management of single-segment thoracic spinal tuberculosis with neurological deficits (STSTND). In this study, the clinical efficacy and feasibility of one-stage posterior-only transpedicular debridement, interbody fusion, and posterior instrumentation for treating STSTND in adults were retrospectively evaluated.

**Methods:**

Thirty-four cases with STSTND underwent one-stage posterior-only transpedicular debridement, interbody fusion and posterior instrumentation at the same institution from January 2003 to January 2013. Follow-up time was 34.4 ± 10.2 months (range, 18–48 months), and kyphosis angle was 34.1 ± 12.3°. The American Spinal Injury Association (ASIA) classification of spinal cord injury was employed to evaluate neurological deficits, while visual analogue scale (VAS) was employed to assess the degree of pain. Erythrocyte sedimentation rate (ESR) and C-reactive protein (CRP) were used to evaluate the activity of tuberculosis (TB).

**Results:**

All 34 patients with spinal tuberculosis (ST) were completely cured, and there was no recurrence of TB. Postoperative kyphosis angle was 8.2 ± 1.8°, and there was no significant loss of correction during the final follow-up. Solid fusion was achieved and pain was relieved in all cases. Neurological condition in all patients improved after surgery.

**Conclusions:**

One-stage posterior-only transpedicular debridement, interbody fusion, and posterior fixation followed by chemotherapy seems to be adequate for obtaining satisfactory healing of single-segment thoracic spinal tuberculosis with neurological deficits. Careful patient selection is critical to the successful outcome with this technique.

## Background

The incidence of tuberculosis is increasing throughout world, especially in developing countries such as China and India. Spinal tuberculosis (ST) is a common extra-pulmonary tuberculosis, and is the most frequent and serious form of skeletal tuberculosis [1, 2]. However, single-segment thoracic spinal tuberculosis with neurological deficits (STSTND) has rarely been reported in literature, except in some case reports in the mainstream academic journals. Although ant-TB chemotherapy and external immobilization remains to play an irreplaceable role in the treatment of ST in most cases, STSTND is characterized by kyphosis deformity, abscess formation and spinal cord compression; which is usually beyond chemotherapeutic function. Therefore, surgical invention would be necessary in these cases. To our knowledge, surgical treatment of STSTND has rarely been reported.

Since anterior radical debridement and non-instrumented fusion was described by Ito and Asami in 1934, followed by Hodgson and Stock in 1956 [3], it has become obvious that even anterior debridement and bone grafting was often unsatisfactory in correcting or preventing the progression of kyphosis deformity [4–7]. High incidence of kyphosis progression has been observed following non-instrumented anterior fusion, compared to non-operative treatment [8]. Some researchers have demonstrated that posterior bone grafting alone had either no evident benefit or was harmful in the presence of active tuberculosis [6, 9]. However, there has been a significant evolution in the treatment of spinal tuberculosis during the past few decades. Posterior pedicle screw system has become popular as a revolutionary technique for correcting angular deformity and stabilizing unstable spines. It has been proven effective for treating many thoracic spinal disorders that result in segmental instability and neurologic impairment [6, 10]. Therefore, the treatment strategy for this disease entity has been revised, which become more conservative and less invasive in recent years.

Posterior instrumentation and fusion, anterior debridement and fusion, and anterior and posterior fusion have been described as effective treatments for ST; but there is a lack of consensus as to the most effective means of managing STSTND. Hence, this study aims to evaluate results/outcomes associated with this approach for the treatment of STSTND in a series of 34 patients.

## Methods

### Inclusion and exclusion criteria and patients

**Fig. 1:**
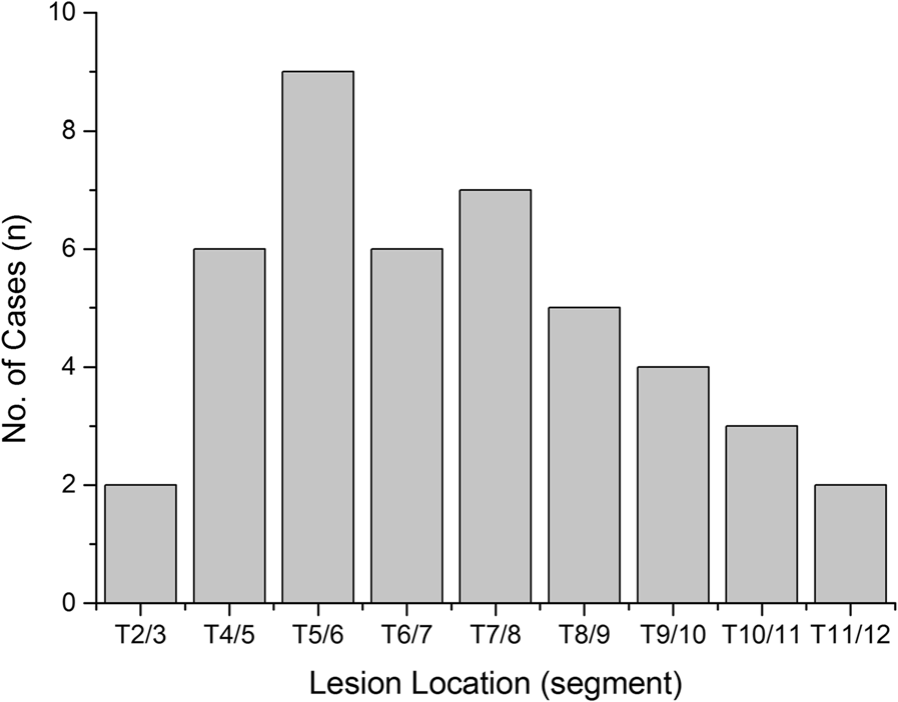
The level of involved thoracic vertebras

Written informed consent was obtained from all patients, and this study protocol was approved by the Ethics Committee of Xiangya Hospital. From January 2003 to January 2013, surgery therapy was performed on 34 patients with STSTND by the same surgeons at the same institution. Among these patients, 20 were male and 14 were female, and patient’s age ranged from 19 to 68 years old (average age, 40.3 years old). Involved levels were observed at two adjacent thoracic vertebras in 30 cases, while three or four vertebras with only one focus center were observed in four cases (Fig. 1). Surgery was considered in the presence of the following indications: (1) persistent back pain that was unresponsive to chemotherapy for two months; (2) progressive neurological deficit and angular deformity (≥30°) or instability is likely to appear; (3) radiologically severe spinal cord decompression (≥1/4 of the spinal canal diameter at the same level), significant vertebra destruction or collapse (≥1/3 of vertebral height), and/or paraspinal abscess with necrotic disc or inflammatory granulation tissues (≤5 cm away from the diseased focus); (4) multilevel vertebrae were involved, in which only one center was required to be debrided and short bone fusion was performed (less than two levels); (5) the elderly or patients with poor health who could not tolerate too much trauma; and (6) patients who underwent anterior operation, in which their anatomical structure was unclear. Patients that presented with the following conditions were excluded: (1) no neurological deficits; (2) multilevel lesions or several focus centers that require anterior long-segment bone fusion; (3) accompanied by paraspinal or deep muscle abscesses beyond its scope and severe kyphosis deformity (more than 50°), in which combined anterior and posterior surgery would be given priority. Diagnosis was based on clinical and hematological criteria. Preoperative kyphosis angle was 34.1 ± 12.3° (range, −10-45°). The American Spinal Injury Association (ASIA) classification of spinal cord injury was employed to assess neurological deficits. Based on ASIA classification, three patients were classified as grade A, five patients were classified as grade B, 15 patients were classified as with grade C, and 11 patients were classified as grade D (Table 1 and Fig. 2). Visual analogue scale (VAS), erythrocyte sedimentation rate (ESR) and C-reactive protein (CRP) of patients upon admission were 7.8 ± 2.1, 40.2 ± 6.3 mm/h, and 22.4 ± 5.7 mg/L, respectively (Table 1).

**Table 1:** Clinical details of surgery group

Schedule	Classification of Neurological function by ASIA					VAS	Kyphosis angle(°)	ESR (mm/h)	CRP (mg/L)
	A	B	C	D	E				
Pre-op	3	5	15	11		7.8 ± 2.1	34.1 ± 12.3	40.2 ± 6.3	22.4 ± 5.7
Post-op		2	4	14	14	4.2 ± 1.8	8.2 ± 1.8	21.3 ± 5.1	10.2 ± 4.5
# TMP		1	3	6	24	2.0 ± 0.5	9.0 ± 1.5	10.5 ± 2.3	4.6 ± 2.0
※ FFU		1	2	4	27	1.4 ± 0.6	9.7 ± 2.0	10.2 ± 3.1	5.0 ± 3.3

*ASIA* the American spinal injury association score system, *VAS* Visual analogue scale of pain, *ESR* erythrocyte sedimentation rate, *CRP* C-reactive protein, *Pre-op* pre-operation, *Post-op* post-operation, *TMP* three months post-operation, *FFU* final follow-up

※, Wilcoxon signed rank test, compare classification of neurological function of pre-operation with final follow-up, *P* < 0.05

#, Paired t test, compared kyphosis angle, VAS, ESR and CRP in 3 month post-operation with pre-operative groups, *P* < 0.05

**Fig. 2:**
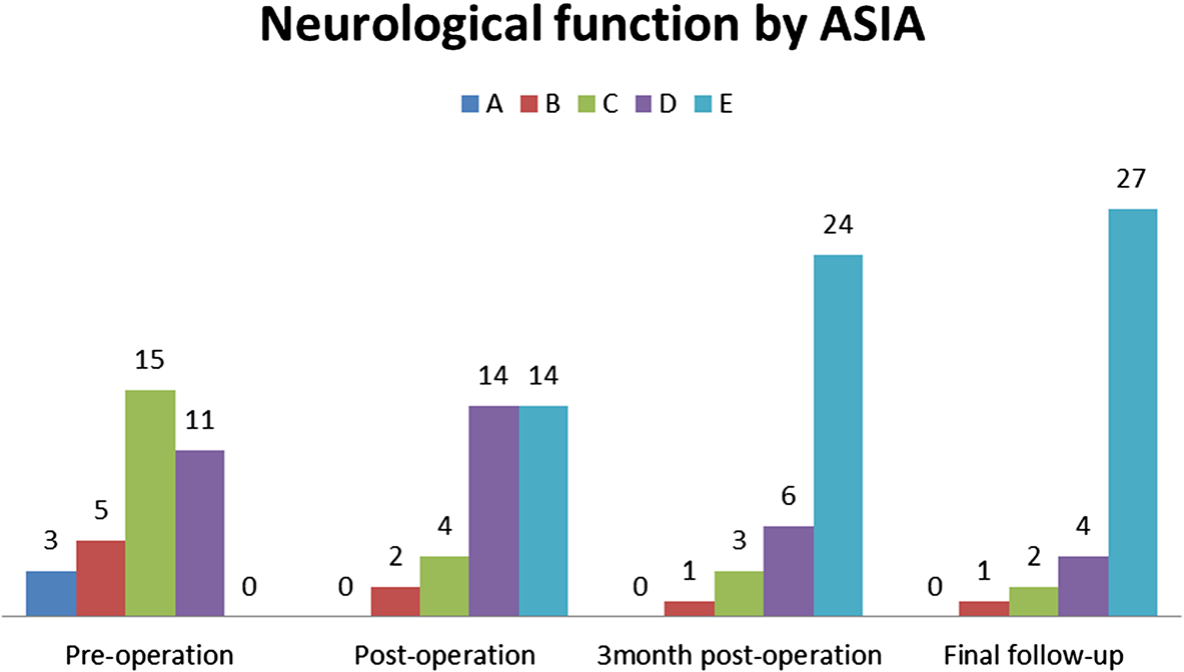
Neurological status of 34 patients. AISA: American Spinal Injury Association

### Preoperative procedure

Chemotherapy was administered shortly after clinical diagnosis was suspected. Anti-TB drugs with HREZ chemotherapy regimen that consisted of isoniazid (5–10 mg/kg/day with no more than 300 mg/day), rifampicin (5–10 mg/kg/day with no more than 300 mg/day), ethambutol (15 mg/kg/day with no more than 500 mg/day), and pyrazinamide (25 mg/kg/day with no more than 750 mg/day) was administered 2–4 weeks before surgery. When progressive neurological deficits appeared and severe back pain was unresponsive to chemotherapy, we appropriately shortened the time of drug treatment. Surgical management was performed when ESR, CRP and temperature returned to normal levels or significantly decreased, and when anemia and hypoproteinemia were rectified completely.

### Operative technique

Patients were placed in the prone position after administering general endotracheal anesthesia. Patients (involving T1-4) were placed in a halo with a weight of 3 kg during the operation. Through a midline incision, posterior spinal elements including lamina, facet joints and transverse processes were exposed (extraperiosteal dissection); extending one vertebrae above and below the involved segments. Transpedicular screws were allowed to be fixed on the side of the vertebral lamina based on preoperative symptoms and imaging. Generally, we preferred a longer segmental fixation that was at least two levels superior and inferior to the level of decompression. Transpedicular screws were also placed in the affected vertebrae if the upper part of the vertebrae was not destroyed by infection. After transpedicular screws were implanted, C-arm X-ray was used to confirm its accuracy. A temporary pre-bent rod was installed on the mild side of the lesion to avoid spinal cord injury induced by instability of the spine during decompression and focal debridement. Then, the severe side of the lesion, which caused clinical symptoms or presented with paraspinal abscess at the decompression side, was selected. A unilateral facetectomy and laminectomy up to the medial pedicle edge were performed. Then, the adjacent rib 1.0–1.5 cm beside the thoracic spine was cut off, and thoracic nerve roots on the focal side were sacrificed for better exposure, if necessary. Generally, decompression range was based on the extent of spinal canal stenosis and the scope of paraspinal abscess. A suitable flush tube was plunged into the paravertebral abscess to wash with appropriate pressure until no pus outflow was observed. Then, the necrotic disc and collapsed vertebrae were removed through to healthy bleeding bone using curettes. When the abscess was beyond the flush tube extension, the patient was repositioned by adjusting the operating table to facilitate the abscess into the focus (postural drainage). Sequentially, rods were tightened, and the kyphosis was slowly and carefully rectified with the help of the compression and stretch of the internal fixation instrument. If the space created after focal debridement was too large, allograft bone would be selected for posterior fusion at the segment that underwent decompression and focal debridement. If necessary, the other side was treated in the same way. If the bone graft was loose or bone defects were present, we embedded relatively large bone particles or blocks into the gap by implementing an impacted graft. Afterwards, 1.0 g of streptomycin and 0.2 g of isoniazid were locally administered, negative pressure drainage and incision sutures were performed postoperatively, and resected specimens were collected for bacterial culture and pathological diagnosis.

### Postoperative care

The drainage tube was pulled out when drainage volume was less 30 ml. Patients continued oral HREZ chemotherapy postoperatively. Six months later, pyrazinamide was discontinued. Then, patients received nine- to 12-month regimens of HRE chemotherapy (6HREZ/9-12HRE) [11]. Ambulation was allowed one week after surgery with a brace. All patients were examined clinically and radiologically in one week, and in three, six and 12 months after surgery; and then, once a year thereafter.

### Follow-up index and statistical analysis

For all cases, the following indexes were recorded preoperatively, postoperatively, three months of postoperation, and during follow-up: (1) kyphosis angle, (2) neurological status, (3) VAS, and (4) ESR and CRP. Using SPSS 19.0 software (SPSS, Inc., Chicago, IL, USA), kyphosis angle, VAS, ESR and CRP were statistically analyzed by paired *t*-test preoperatively, postoperatively, three months of postoperation, and during follow-up; while neurological function was statistically analyzed by Wilcoxon signed rank test preoperatively, postoperatively, three months of postoperation, and during follow-up. Discrepancy of normal distribution was analyzed by rank-sum test with a significance level of 0.05.

## Results

### Basic condition

Follow-up duration was 34.4 ± 10.2 months, operation time was 152.1 ± 24.4 minutes, blood loss was 650.7 ± 150.2 ml, and hospitalization time was 12.4 ± 4.1 days (Table 2). Some complications occurred after operation such as cerebrospinal fluid leakage (four cases), water-electrolyte imbalance (10 cases), superficial infection (two cases), and mild intestinal obstruction (five cases). No complication related to the grafted bone and instrumentation was observed postoperatively, and symptoms disappeared after the patient underwent anti-inflammatory or symptomatic supportive treatment for 1–2 weeks.

**Table 2:** General data of study

Gender		Age (years)	follow-up time(mon)	Operation time (min)	Amount of bleeding (ml)	Hospitalization day (days)	Bone fusion time (mon)
Male	Female						
20	14	40.4 ± 11.2	34.4 ± 10.2	152.1 ± 24.4	650.7 ± 150.2	12.4 ± 4.1	4.5 ± 3.2

### Neurologic function and pain

Neurologic deficits in all patients improved at the final follow-up examination. Results were evaluated by ASIA classification during the final follow-up, as follows: one case improved by three grades, 21 cases improved by two grades, and 12 cases improved by one grade (Fig. 2 and Table 1). Statistical analysis revealed that there was a significant difference between pre-operation and the final follow-up (*P <* 0.05). Seven patients revealed incomplete neurological function during the final follow-up, and attributed to delayed diagnosis. VAS of pain was 7.8 ± 2.1 preoperatively, which dropped to 4.2 ± 1.8 postoperatively and 1.4 ± 0.6 during the final follow-up. All patients had no recurrence of TB, and all patients had pain relief.

### Kyphosis deformity, bone fusion, ESR and CRP

**Fig. 3:**
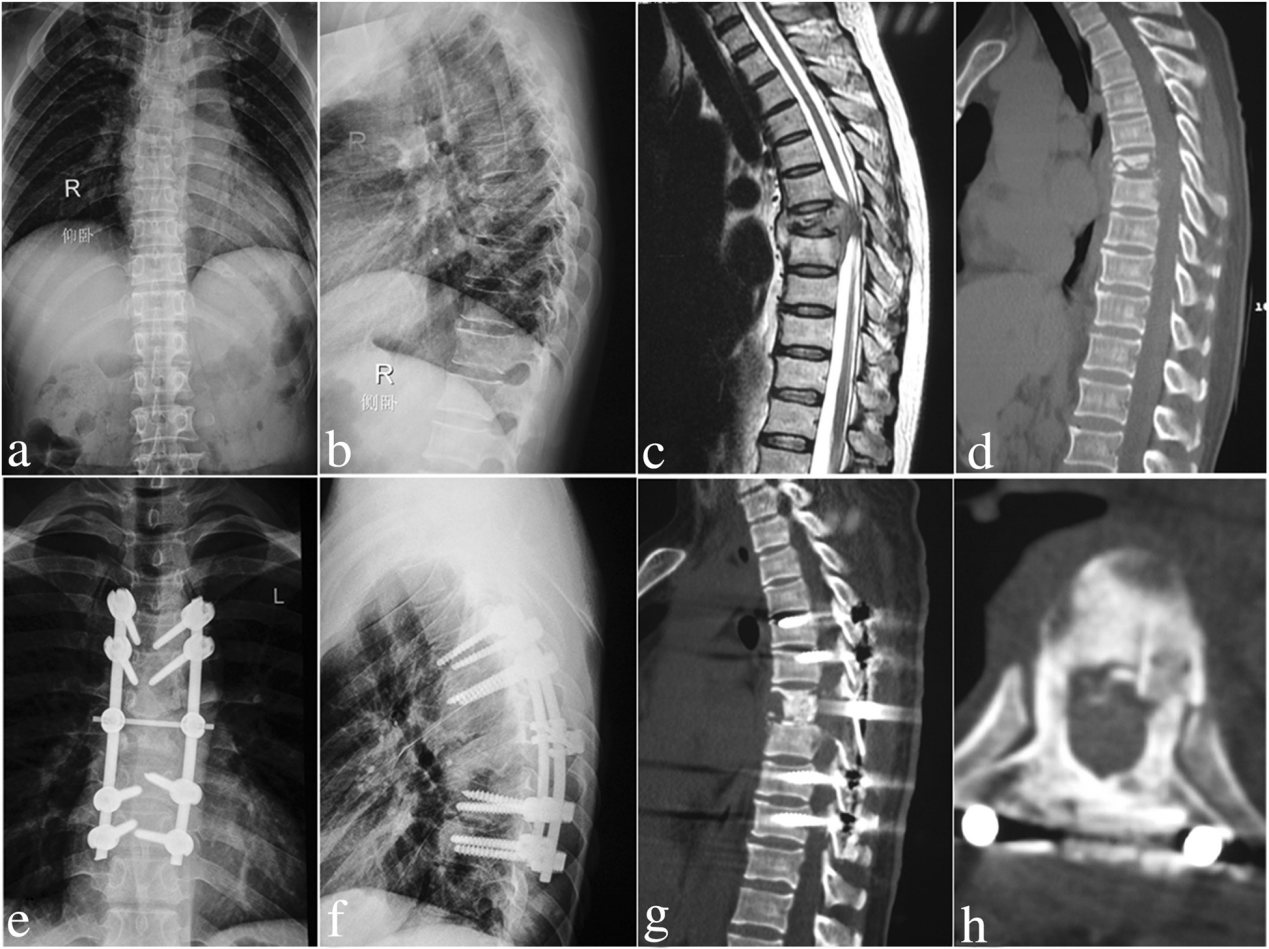
A 52-year-old female with T5/6 lesions was performed by posterior-only approach. **a**-**d** The pre-operative imaging data showed T5/6 vertebral bodies’ destructions with mild kyphosis deformity and spinal cord severely compressed. The postoperative anterior-posterior (**e**) and lateral X-ray (**f**) indicated that the kyphosis got obviously improved by posterior long-segment fixation. Sagittal and coronal CT-scan (**g**, **h**) showed satisfied allograft fusion without relapse of Pott’s disease at the 9 months of post-operation

**Fig. 4:**
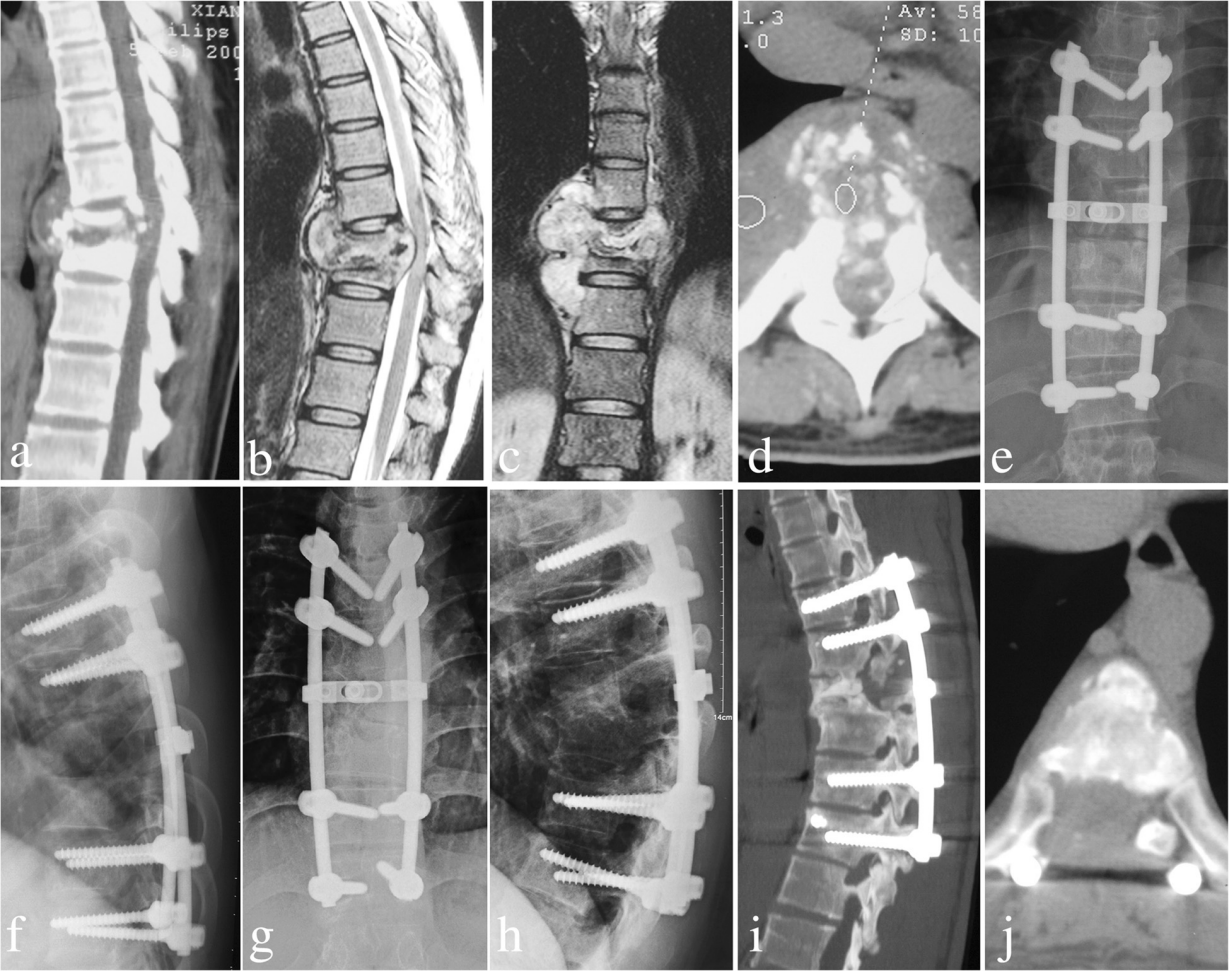
A 27-year-old male with T8/9 lesions was performed by posterior-only approach. **a**-**d** The pre-operative imaging data showed T8/9 presented with severe bone destruction, formation of local paraspinal abscess and compression of spinal cord. The postoperative anterior-posterior (**e**) and lateral X-ray (**f**) indicated posterior long-segment fixation was in good position. X-ray (**g**, **h**) showed good bone fusion and no obvious loss of kyphosis angle in 3 months postoperative. Sagittal and coronal CT-scan (**i**, **j**) showed satisfactory bone fusion without presence of complications related to instrumentation and relapse of Pott’s disease at the final follow-up

Kyphosis angle was 34.1 ± 12.3°, preoperatively; which significantly decreased to 8.2 ± 1.8°, postoperatively (*P* < 0.05). Kyphosis angle was 9.7 ± 2.0° at final follow-up with a loss of correction of only 1.5 ± 0.6°. This continued to significantly improve compared to preoperative measurements (*P* < 0.05). Intervertebral bone graft and intertransverse fusions were performed in all patients. Lateral X-ray or CT was used to assess the fusion and formation of the bone bridge. All patients achieved bone fusion within 4.5 ± 3.2 months after surgery, which was confirmed by two different surgeons based on the modified criteria of Lee *et al.* [12] for radiological fusion (Figs. 3 and 4). Average pretreatment for ESR and CRP was 40.2 ± 6.3 mm/h and 22.4 ± 5.7 mg/L, respectively; which returned to normal levels during the final follow-up in all patients. There was a statistical difference in ESR and CRP between the preoperative period and during final follow-up (*P* < 0.05). (Table 1)

## Discussion

Tuberculous spondylitis is a frequently occurring disease that accounts for approximately 50 % of bone and joint tuberculosis [13]. Formal and sufficient anti-TB chemotherapy, strict bed rest, and supportive therapy are the most basic methods for treating ST. However, conservative regimes would yield to surgical approaches for patients predisposed to bone destruction, sequestrum formation, paraspinal abscess and nerve compression. Consequently, surgical indications of STSTND should be appropriately lowered due to neurologic impairment; and late-onset paraplegia should be given priority. However, there are quite a few controversies on the surgical management of STSTND. Some researchers insist that the anterior approach allows direct access to the focus through debridement and convenient bone grafting [9, 14–17]. However, the high frequency of pseudarthrosis, ineffective correction of kyphosis and maintenance of the correction, and unsatisfactory neurological function overwhelm its advantages. Moreover, the anterior exposure of the upper thoracic spinal region blocked by thoracic bones, as well as the clavicle, costal bone and superior mediastinum organs, presents a significant challenge to spinal surgeons, especially when destruction by infection leads to kyphosis [6, 18, 9, 12]. Combined anterior and posterior surgery has become popular due to its perfect clinical outcome [9, 19]. However, when the aged are complicated by poor condition, it would be difficult to tide over the terrible trauma such as greater loss of blood, longer operation time, and complications related to the anterior approach [4, 6, 19]. Furthermore, there have been relatively few literature reports in the surgical management of STSTND via one-stage posterior transpedicular thoracic debridement, interbody bone grafting, and posterior fixation.

The anterior column is prone to be affected by *Mycobacterium tuberculosis*. Controversies on the application of a posterior only surgery in treating STSTND focus on whether surgeons can completely perform focal debridement and anterior decompression on the circumstance of a limited visual field, whether it would affect the stability of the spine, and whether it would affect the anterior bony fusion [20]. The following characteristics of the posterior approach can solve the above doubts.

1. Stand-alone posterior surgery creates enough operating space through the resection of both sides of the facet joint, diapophysis, lamina and nerve root; which allows the vertebral body to be operated with a 360-degree angle under the direct visualization of the outer layer of the dura mater for the thorough removal of the sequestrum, collapsed vertebras, and intervertebral disk, as well as complete spinal decompression, without injuring the spinal cord. This helps avoid possible intra- and post-operative complications that may be associated with the exposed and debrided anterior (Fig. 2). Paraspinal abscess, if present, would be conquered by appropriate pressure washing and postural drainage. It would certainly be a good choice for some scholars to promote the removal of lesions by a thoracoscope [21]. In our study, all patients had no recurrence of TB. It has been queried that removing the TB focus via the posterior approach could cause spinal cord infection and central nervous system complications such as TB meningitis. However, in our study, none of the patients was complicated by TB meningitis; and this finding is consistent with other reports [5, 6].
2. Based on the scope of vertebral destruction and extent of vertebral osteoporosis, surgeons were allowed to have multiple points of fixation along the spinal axis as opposed to anterior plating, which relies solely on endpoint fixation [7]. Furthermore, thorax support is essential for effective load transferability. However, internal fixation is likely to cause fatigue damage, and may lead to screw and rod loosening or fracture. Thus, it is necessary to implant autologous or allograft bone in the lateral facet joints of the diseased vertebrae and between the transverses, in order to provide bone support for spine stability after interbody fusion [5].
3. ST is prone to involve the anterior column of a single motion segment such as two adjoining vertebral bodies and their intervening disc (peridiscal). Therefore, inserting bone grafts into the anterior and central column of the spine can play a major role of resisting vertical compressive stress, torsional force and shear force from the spine; and in sharing the partial load of the internal fixation system. This prevents excessive stress to focus on the internal fixation screws, and avoid the emergence of kyphosis recurrence and late-onset paraplegia. Mutually, some pressures from the internal fixation at the graft-endplate interface would serve as very relevant biomechanical indicators of biological phenomena such as bone fusion [22]. Therefore, bone fusion combined with instrumentation was in full compliance with the requirements of the biomechanics of the spine. However, residual inter-body distance has a direct impact on the survival rate of the bone graft after debridement [23]. The difficulty and key in the posterior-only surgical treatment of STSTND lies on the processing of relatively long inter-vertebral defects, which would seriously affect the stability and support functions of the spine. In addition, a long-segment bone graft is more prone to delayed stress fractures, leading to severe loss of correction of kyphosis. Thus, compressive stress that form the upper and lower ends of the vertebras are concentrated on the screws, causing these screws to loosen and break; which result in further kyphosis and late-onset paraplegia [24]. Therefore, we reserved the viable bone tissue as much as possible, and inserted an appropriately trimmed graft into the inter-vertebral disc space. If the graft bone was loose or bone defects are present, we embedded relatively large bone particles or blocks into the gap by implementing an impacted graft. Danis [25] once described in 1953 that impacted graft incorporation was conducive to play a supporting role in spinal stability, and even has beneficial effects for bone fusion. In our study, all patients achieved bone fusion within 4.5 ± 3.2 months after surgery; and there was no complication related to bone fusion. During the operation, we scraped the surface of the sclerotic bone up to the bleeding sub-healthy bone tissue without complete resection, performed an all bone graft, and applied an allogeneic bone rather than an autologous iliac bone to avoid donor-site complications such as pain, infection and unhealed wounds.

The authors consider that the methods reported above are radical. Therefore, the following points should be emphasized when adopting these methods: (a) effective chemotherapy is available at present to sterilize *Mycobacterium tuberculosis* without the need for aggressive anterior debridement; (b) complications related to the anterior approach to the thoracic spine can be avoided; (c) posterior instrumentation can fully serve to correct angular deformity and minimize loss of correction, combined with posterior interbody and posterolateral intertransverse fusion; (d) multilevel vertebrae that involve only one center require short bone grafting fusion (less than two levels), and it is unnecessary to obtain debridement in each lesion; (e) carefully study preoperative radiological imaging and CT scans to determine the feasibility of installing screws, because pedicle structure and bone quality would also affect the placement of screws; (f) application of intraoperative electrophysiological monitoring such as computer navigation monitoring systems, so that some subjective factors can be ruled out; (g) ensure the security of the spinal cord during debridement, decompression and interbody fusion; (h) master postural drainage technology during the operation, and place the drainage tube after the operation to treat the abscess.

## Conclusion

In summary, one-stage posterior transpedicular debridement, interbody fusion and posterior instrumentation are feasible and effective for treating STSTND. However, careful patient selection is critical to obtaining a successful outcome with these techniques. More centers that involve anterior long-segment bone fusion or multiple involvements, in which combined anterior and posterior approaches are necessary, are required. Further, a larger number of patients and longer follow-ups would be required.

## Abbreviations

STSTND: Single-segment thoracic spinal tuberculosis with neurological deficits
ASIA: The American Spinal Injury Association score system
VAS: Visual analogue scale
ESR: Erythrocyte sedimentation rate
CRP: C-reactive protein
TB: Tuberculosis
ST: Spinal tuberculosis
CT: Computed tomography
